# Comparative Transcriptome Analyses during the Vegetative Cell Cycle in the Mono-Cellular Organism *Pseudokeronopsis erythrina* (Alveolata, Ciliophora)

**DOI:** 10.3390/microorganisms8010108

**Published:** 2020-01-12

**Authors:** Yiwei Xu, Zhuo Shen, Eleni Gentekaki, Jiahui Xu, Zhenzhen Yi

**Affiliations:** 1 Guangzhou Key Laboratory of Subtropical Biodiversity and Biomonitoring, School of Life Science, South China Normal University, Guangzhou 510631, China; 2 Pilot National Laboratory for Marine Science and Technology (Qingdao), Qingdao 266237, China; 3 Institute of Microbial Ecology & Matter Cycle, School of Marine Sciences, Sun Yat-sen University, Zhuhai 519000, China; 4 Southern Marine Science and Engineering Guangdong Laboratory (Zhuhai), Zhuhai 519000, China; 5 School of Science, Mae Fah Luang University, Chiang Rai 57100, Thailand

**Keywords:** ciliate, eukaryotes, RT-qPCR, unicellular transcriptome, vegetative cell cycle

## Abstract

Studies focusing on molecular mechanisms of cell cycles have been lagging in unicellular eukaryotes compared to other groups. Ciliates, a group of unicellular eukaryotes, have complex cell division cycles characterized by multiple events. During their vegetative cell cycle, ciliates undergo macronuclear amitosis, micronuclear mitosis, stomatogenesis and somatic cortex morphogenesis, and cytokinesis. Herein, we used the hypotrich ciliate *Pseudokeronopsis erythrina*, whose morphogenesis has been well studied, to examine molecular mechanisms of ciliate vegetative cell cycles. Single-cell transcriptomes of the growth (G) and cell division (D) stages were compared. The results showed that (i) More than 2051 significantly differentially expressed genes (DEGs) were detected, among which 1545 were up-regulated, while 256 were down-regulated at the D stage. Of these, 11 randomly picked DEGs were validated by reverse transcription quantitative polymerase chain reaction (RT-qPCR); (ii) Enriched DEGs during the D stage of the vegetative cell cycle of *P. erythrina* were involved in development, cortex modifications, and several organelle-related biological processes, showing correspondence of molecular evidence to morphogenetic changes for the first time; (iii) Several individual components of molecular mechanisms of ciliate vegetative division, the sexual cell cycle and cellular regeneration overlap; and (iv) The *P. erythrina* cell cycle and division have the same essential components as other eukaryotes, including cyclin-dependent kinases (CDKs), cyclins, and genes closely related to cell proliferation, indicating the conserved nature of this biological process. Further studies are needed focusing on detailed inventory and gene interactions that regulate specific ciliated cell-phase events.

## 1. Introduction

The cell cycle is a series of processes during the life history of a cell that lead to growth and proliferation [1]. During its life history, a cell undergoes multiple rounds of division. Studying mechanisms of cell cycles, of which proliferation is a major part, is important for understanding cell cycle regulation, development, and cell biology [2,3,4,5]. In recent decades, cell cycles of different organisms have been examined in detail using a variety of molecular methods. For instance, cell division cycle-modulated genes in yeast [6], human cells [7], plant cells [8], and ciliated protozoa [9] have been identified and characterized using microarrays, cDNA-amplified fragment length polymorphism (AFLP), or deep RNA sequencing (RNA-Seq). Reverse transcription quantitative polymerase chain reaction (RT-qPCR), the “gold” standard method for mRNA quantification [10], has also been used to explore gene expression during the cell cycle [11]. Nonetheless, using any of these methods to investigate the molecular mechanisms of the cell cycle requires large numbers of synchronous cells [8]. However, this is nearly impossible to accomplish for most species of unicellular eukaryotes. Recent investigations have shown that results obtained from RNA-Seq and RT-qPCR analyses of single cells were comparable to those obtained from bulk samples [12,13]. Thus, these technologies can be used to study the gene expression and cell cycle regulation of unicellular eukaryotes. The cell cycle of unicellular eukaryotes is of interest because the process is inexorably linked to cell division in these organisms.

**Figure 1:**
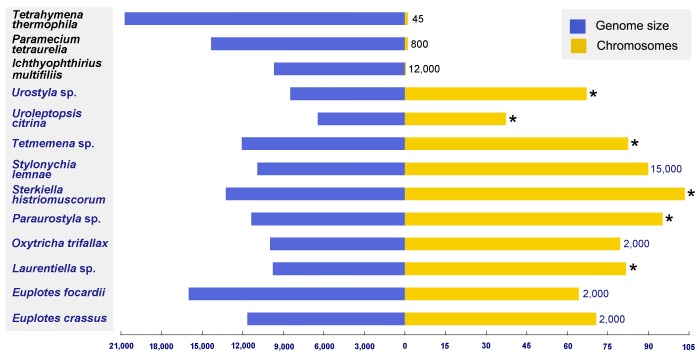
Comparison of 13 ciliate macronuclear genomes. Blue lines denote the genome size; yellow lines represent the number of chromosomes. Species names are listed on the left, while macronuclear ploidy is shown on the right. Asterisks denote that macronuclear ploidy is unclear. Species names and ploidy are depicted in different colors for different ciliate groups (Spirotrichea: dark blue; Oligohymenophorea: black). All data were obtained from references [20,28,29,30,31,32,33,34,35,36,37].

Ciliates, a group of unicellular eukaryotes, have been used extensively as model organisms to study a broad range of topics due to dual genome architecture, unique macronuclear genome characters (Figure 1), and complex morphology [14,15,16,17,18,19,20,21,22,23,24]. Recently, molecular events underlying the sexual cell cycle of the model ciliate *Tetrahymena thermophila* were examined. Using microarray and high-throughput sequencing, Xiong et al. [9] and Miao et al. [25] identified genes that were specifically up- or down-regulated in growing, starved, and conjugating cells. Knocking out the CYC2 cyclin gene revealed its crucial role in *T. thermophila* meiosis [26]. In a later study, Xu et al. [27] further researched the Cyc2p function and its precise regulation mechanism during the micronuclear elongation of *T. thermophila*.

Vegetative proliferation is asexual cell division in ciliates and occurs by binary fission. The vegetative cell cycle consists of three consecutive periods, i.e., growth (G), morphogenesis (S), and cell division (D) [14]. Two “sister cells”—the proter and the opisthe—are produced during the process of cell division, which occurs after duplication of organelles (cell morphogenesis). Morphogenetic studies have revealed rich morphological patterns during the process [38,39,40]. This is due to a multitude of events that occur during that time including macronuclear amitosis, micronuclear mitosis, cortical stomatogenesis, somatic cortex morphogenesis, and cytokinesis [14]. Molecular mechanisms underlying vegetative cell division of ciliates are largely unknown, with the exception of a study showing that Ca^2+^/CaM, p85, and RAN have important roles in the progression of cell division in *Tetrahymena* [40,41]. Ciliate cell division is more complex than that of other eukaryotes, comprising at least 4 types and 15 modes of stomatogenesis, and hence is of great significance for understanding the evolution of this process in eukaryotes [42].

Herein, we used the hypotrich *Pseudokeronopsis erythrina* to examine the molecular mechanisms of its vegetative cell division cycle. This is an atypical ciliate species with apokinetal stomatogenesis, whereby proliferation of kinetosomes occurs independently of the parental oral apparatus [15]. The vegetative cell cycle of this genus has been well investigated in morphogenetic studies [15,43]. Furthermore, the large cell size of *P. erythrina* (200 vs. 50 μm in *Tetrahymena*) is conducive to accurately identifying different stages of the vegetative cell cycle when using microscopy. Single-cell RNA-Seq and RT-qPCR methods were applied to identify key pathways and genes underlying vegetative cell division.

## 2. Materials and Methods

### 2.1. Cell Growth and Sorting

**Figure 2:**
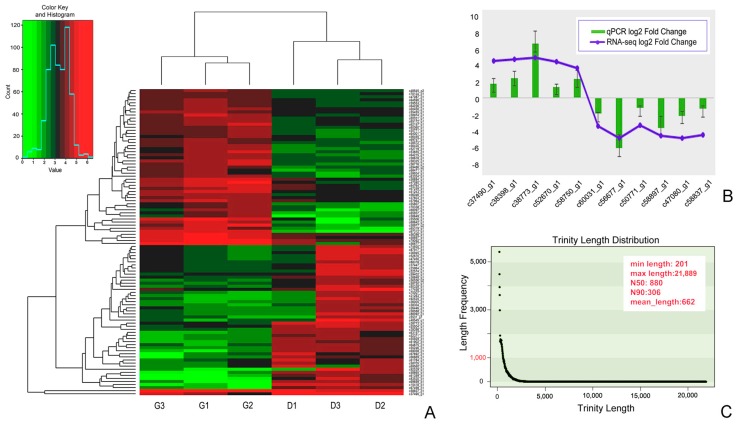
Statistics of transcriptomic data and cluster analysis of differentially expressed genes (DEGs). (**A**), Heatmap of the top 100 DEGs between the cell division (D) and growth (G) stages from three replicates. Each column represents a sample, and each row represents a unigene. (**B**), Relative transcription levels of DEGs in group D in comparison with group G shown by RT-qPCR and RNA-Seq. Blue lines represent the fold change of gene transcription revealed by RNA-Seq using log2. Green bars represent the relative transcription level determined by RT-qPCR using log2 (2^−ΔΔct^). Error bars represent standard deviations from three independent biological replicates. (**C**), Length distributions of unigene sequences derived from the transcriptome assembly of *Pseudokeronopsis erythrina*.

*Pseudokeronopsis erythrina* cells were sampled from the Pearl River estuary (22°41′ N; 113°38′ E), Guangdong, China, and then cultured at room temperature in artificial seawater with a rice grain to enrich the growth of bacteria [44]. We picked one cell at the cell division (D) stage in morphogenesis for each replicate and two individual cells at the growth (G) stage for each replicate, considering that cell volume at the D stage is nearly double that of a cell at the G stage (Figure 2A). The latter originated from the same cell 30 minutes after cell division. In order to reduce cell heterogeneity, three replicates were collected for each stage: G1, G2, G3 and D1, D2, D3. Each cell was washed five times with inactivated calcium and magnesium-free PBS buffer using a nuclease-free pipette and was then transferred to a nuclease-free Eppendorf tube with a minimum volume of liquid.

### 2.2. Single Cell cDNA Amplification and Library Construction

Each cell was placed into a tube containing 2 μL of cell lysis buffer (0.2% Triton X-100 and 2U μL/1 RNase inhibitor) and kept in a volume as low as possible (less than 5 μL). Collected cells were amplified using the Smart-Seq2 of SMARTer Ultra Low RNA Kit for Illumina sequencing [45]. The cDNA concentration was measured using a Qubit 3.0 Fluorometer (Life Technologies, Foster City, CA, USA).

### 2.3. Sequence Assembly and Analysis

Each single-cell library was sequenced (1 × 50 bases) using an Illumina HiSeq 2000 with replicate libraries of each type in the same lane. Raw reads were processed with the existing sequence grooming tool FastQC, assembled with Trinity v.2.1.1 [46] and aligned using Bowtie2 v2.2.3 [47]. Q30, N50, the GC content, and the sequence duplication level of clean data were calculated. The unigenes were annotated based on the following six databases: NCBI non-redundant protein sequence (nr) database, NCBI nucleotide sequence (nt/nr) database, PFAM, Clusters of Orthologous Groups (COG), Kyoto Encyclopedia of Genes and Genomes (KEGG), and Gene Ontology (GO), using BLAST with a cut off e-value of < 10^−5^. In order to ensure that all annotated genes were ciliate specific, we selected ciliates genes against annotated genes in the nr database using BlastX.

### 2.4. Differential Gene Expression

Expression levels were estimated as follows: cleaned reads were mapped back onto the assembled transcriptome and then the read count for each predicted gene was obtained from the mapping results and was normalized to reads per kb of exon model per million mapped reads (RPKM). Differential expression analysis of two samples was performed using DEGseq2 [48]. The *p*-value was adjusted using the *q*-value, which was defined as the multiple testing analog of *p*-value, and *q*-value < 0.05 and |log2 fold change (FC)| ≥ 1 were set as the threshold for differential expression. Variations in predicted gene expression levels were analyzed for specific comparisons that encompass two categories: (i) cells at the D stage and (ii) cells at the G stage. GO terms and KEGG pathway enrichment were used to analyze differentially expressed genes (DEGs) using the online tool OmicShare (http://www.omicshare.com/tools/). The threshold of the false discovery rate (FDR) was set at 0.05. For each group, the Euclidean distance was calculated according to the expression level of DEGs, taking the logarithm of the base 10, and then hierarchical clustering was performed using OmicShare tools to obtain the overall clustering results for each group.

### 2.5. Reverse Transcription Quantitative Polymerase Chain Reaction (RT-qPCR)

Cells of *Pseudokeronopsis erythrina* were collected as described above. Total RNA was extracted with an RNeasy Plus Micro Kit (Qiagen, Hilden, Germany). cDNA was generated using SuperScript^®^ III Reverse Transcriptase (Life Technologies, Carlsbad, CA, USA). RT-qPCR primers were designed using Primer Premier 5.0 software (PREMIER Biosoft International, Palo Alto, CA, USA) and Primer-BLAST (http://blast.ncbi.nlm.nih.gov/) (Supplementary Table S1). RT-qPCR was performed on an Applied Biosystems^®^ QuantStudio^®^ 5 instrument (Applied Biosystems, Carlsbad, CA, USA), and the reaction was conducted in a 20 μL reaction system containing 10 μL of the QuantiNova SYBR Green PCR kit (Qiagen, Hilden, Germany), 10 μM of each primer, cDNA and nuclease-free water (Qiagen, Hilden, Germany). PCR cycling conditions were set as follows: 2 min at 95 °C, 40 cycles of 5 s at 95 °C, and 10 s at 60 °C. Three biological replicates were used for all experiments and for each biological replicate, and three technical replicates were employed [49]. The relative expression of each predicted gene was calculated by the comparative 2^−ΔΔct^ method [50] with RpS6 used as the housekeeping gene [51].

## 3. Results

### 3.1. Transcriptome Sequencing, Assembly Evaluation and Annotation of Unigenes

In total, 297,955,888 raw reads were obtained from six transcriptomes. After removing low-quality regions, adapters, and contaminants as much as possible, we obtained clean reads with numbers ranging from 35,788,890 to 43,051,906. Reads have been deposited in GenBank under BioSample number SAMN13668481-SAM13668486 with Bio Project ID PRJNA597169. Clean reads with a quality score Q30 comprised 81.4% of raw reads (Table 1). The overall GC content of contigs was 43.83% and that of unigenes was 44.59%. The total length and number of contigs were 58,260,420 bp and 88,014, respectively (Supplementary Table S2). The maximum contig length was 21,889 bp with an average length of 661.94 bp (N50: 880). The total number of unigenes was 76,358 comprising 47,567,028 bp. The longest unigene was 21,889 bp, while the average length was 622.95 bp (N50: 806). The length distribution of unigene sequences is shown in Figure 2C.

Of the 76,358 unigenes, 19,884 (26%) had a best hit to genes of ciliates in the NCBI non-redundant protein sequence (nr) database and were used for downstream analyses. Of these, 5489 unigenes were aligned to the COG database, 7730 unigenes were annotated with GO functions, and 4354 unigenes were mapped using KEGG.

### 3.2. Differentially Expressed Genes between the G and D Stages

Reads per kb of exon model per million mapped reads (RPKM) results showed that 2051 unigenes were differentially expressed (with fold changes > 2 and *q* < 0.05) between groups G and D. Among these unigenes, 1545 were up-regulated, while 506 were down-regulated during the D stage (Supplementary Table S3). Most cyclin, anaphase-promoting complex (APC), and cyclin-dependent kinase (CDKs) genes were up-regulated. Other mostly up-regulated unigenes were dual-specificity tyrosine-(Y)-phosphorylation regulated kinase (DYRK), MORN domain-containing proteins, and mitogen-activated protein kinases (MAPK), all of which are known for their involvement in the cell cycle (Table 2).

**Figure 3:**
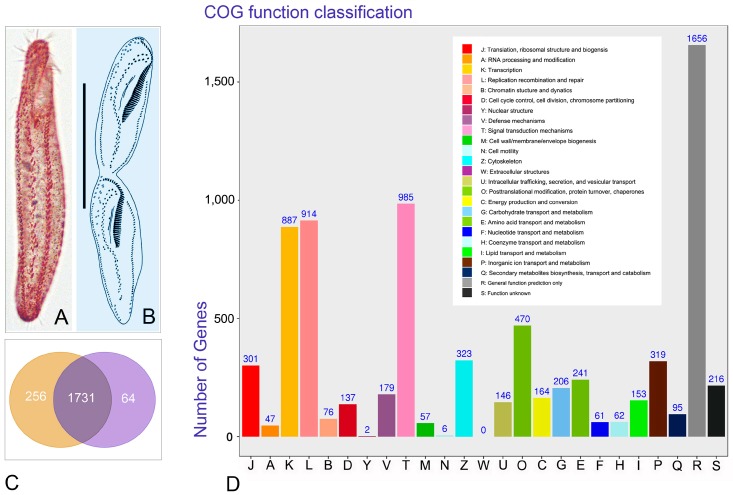
Morphology of *Pseudokeronopsis erythrina*, analysis, and functional annotation of unigenes. (**A**,**B**), *P. erythrina* from life at growth stage (**A**) and infraciliature at cell division stages from Chen et al. [15] (**B**). (**C**), Venn diagram of DEGs for stage D and stage G. Unigenes shared by stages D and G are in dark purple, while unique ones at stage D and G are in orange and light purple, respectively. (**D**), Annotation of unigenes according to clusters of orthologous groups (COG). Numbers above bars represent predicted unigenes. Scale bars in (**A**, **B**): 60 μm.

Among 256 uniquely expressed unigene during stage D (Figure 3A–C), 140 (54.69%) had known functions in the nr database, while 116 (45.31%) were hypothetical protein genes with unknown functions. At the G stage, 64 unigenes were uniquely expressed (Figure 3A–C), 42 of which (65.63%) had known functions in the nr database, while 22 (34.37%) were hypothetical protein genes and with unknown functions.

### 3.3. Annotation of Unigenes Using COG Functional Categories

A total of 5489 unigenes had a COG classification covering 24 functional classifications (Figure 3D). The top three identified functional categories were as follows: general function prediction only accounted for 30.17% (*n* = 1656); signal transduction mechanisms for 17.94% (*n* = 985), and replication, recombination, and repair for 16.65% (*n* = 914). There were 482 DEGs between groups D and G, of which 331 were up-regulated and 151 down-regulated. In each case, DEGs covered 22 COG categories (Supplementary Table S4). It is noteworthy that 19 significantly transcribed DEGs were enriched in cell cycle control, cell division, and the chromosome partitioning category (Supplementary Table S5) with a single unigene being down-regulated, while all others were up-regulated. Up-regulated unigenes mainly contained kinase and cyclin genes.

### 3.4. Annotation of Unigenes Using GO Enrichment

A total of 7730 unigenes were assigned to three major functional GO terms: 82.90% (*n* = 6408) of unigenes were assigned to the biological process level, and 85.74% (*n* = 6628) and 86.46% (*n* = 6683) of unigenes were assigned to the cellular component and molecular function level, respectively. Within the biological process level, most abundant categories were cellular, single-organism, and metabolic process. The GO terms cell, cell part, and organelle were most abundant with respect to the cellular component category. Within the molecular function level, unigenes were predominantly associated with binding, catalytic activity, and transporter activity terms. These unigenes were further summarized into 60 sub-categories using Blast2GO.

**Figure 4:**
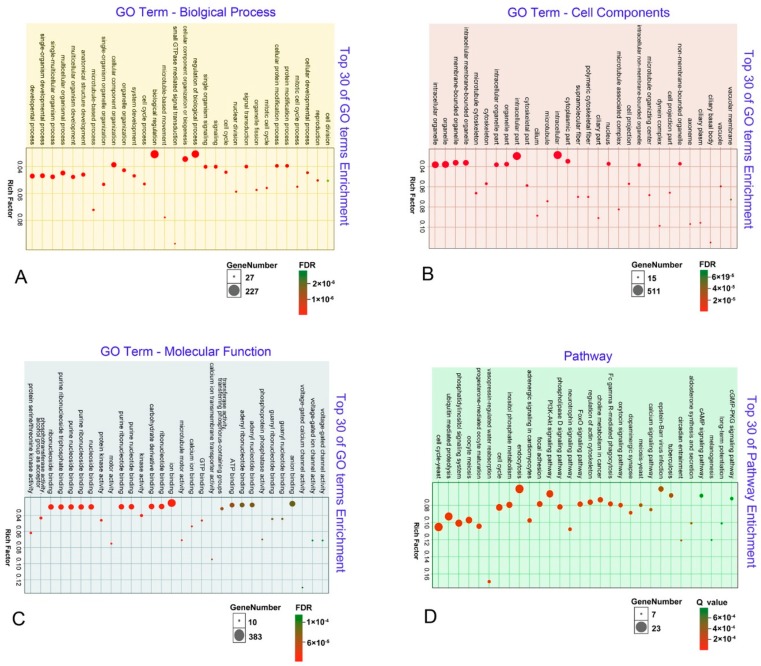
Top 30 GO terms and enrichment of the DEGs at the D stage in the biological process category (**A**), cell components category (**B**), molecular function category (**C**), and KEGG pathway enrichment (**D**). The size of dots indicates the number of DEGs. The color of dots corresponds to the FDR/Q value.

Within the 7730 unigenes, 725 were DEGs between group D and G. Of these, 151 were significantly enriched in the biological process category with the top ones being developmental processes, including single-organism developmental and single-multicellular organism processes (Figure 4A). Other significant processes included microtubule-based process, cilium morphogenesis, development, and cell division (Supplementary Table S6). Thirty-five DEGs were enriched in stage D, of which 28 were up-regulated and 7 were down-regulated. Up-regulated DEGs mainly contained cyclin and cyclin-dependent kinase (Supplementary Table S3). In the cell components category, there were 92 significantly enriched GO terms (Supplementary Table S6). The top five significant GO terms were intracellular organelle, organelle, membrane-bound organelle, intracellular membrane-bound organelle, and microtubule cytoskeleton. Significant pathways included the nucleus, ciliary basal body, and anaphase-promoting complex (Figure 4B). There were 90 significantly enriched GO terms in the molecular function category (Supplementary Table S6) including protein serine/threonine kinase activity, phosphotransferase activity, and ribonucleoside binding (Figure 4C). The top three pathways were the yeast cell cycle, ubiquitin-mediated proteolysis, and the phosphatidylinositol signaling system (Figure 4D).

### 3.5. Annotations of Unigenes Using KEGG

**Figure 5:**
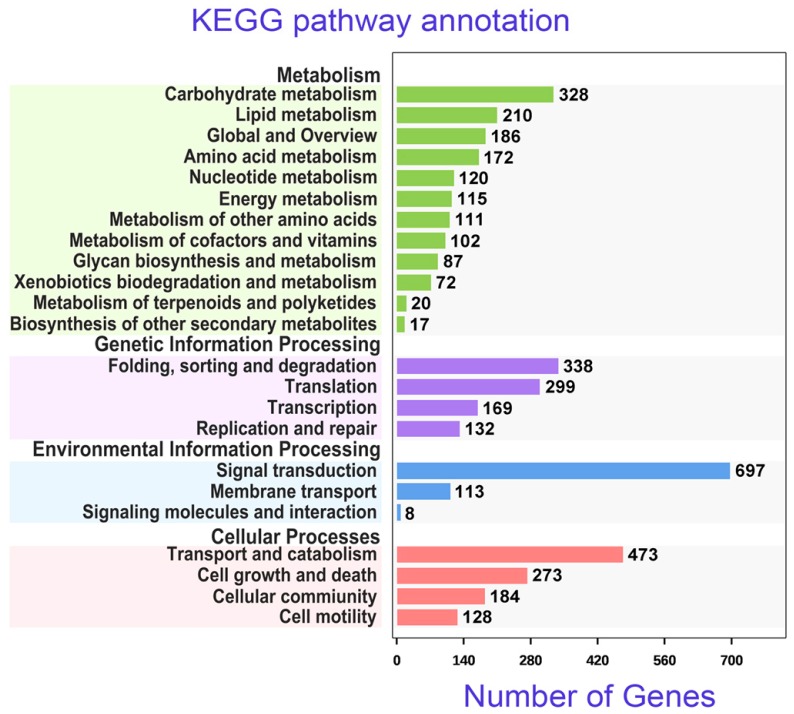
Number of unigenes in *Pseudokeronopsis erythrina* annotated in KEGG pathways.

In sum, 4354 unigenes of *Pseudokeronopsis erythrina* mapped onto KEGG pathways that were related to metabolism, genetic information processing, environmental information processing, and cellular processes (Figure 5). Of these unigenes, 489 DEGs were mapped to 129 pathways. Fourteen enriched pathways (*q*-value < 0.05) were mainly related to cell growth and death, as well as signal transduction, when comparing group D with group G (Supplementary Table S7). Anaphase-promoting complex subunit 11, anaphase-promoting complex subunit 8-like, anaphase-promoting complex subunit 10, and the TRR repeat-protein, closely related to cell division, were involved in the four most significant KEGG pathways.

### 3.6. RT-qPCR Validation of DEGs

A heatmap of the 100 most highly expressed DEGs showed 51 down-regulated genes in the top branch, 47 up-regulated genes in the next branch, while the nethermost branches contained only two genes (Figure 2A). There were two distinct clusters at the left and right sides of the heatmap. DEGs of groups G and D were clearly separated, with samples from individual stages grouping together.

**Figure 6:**
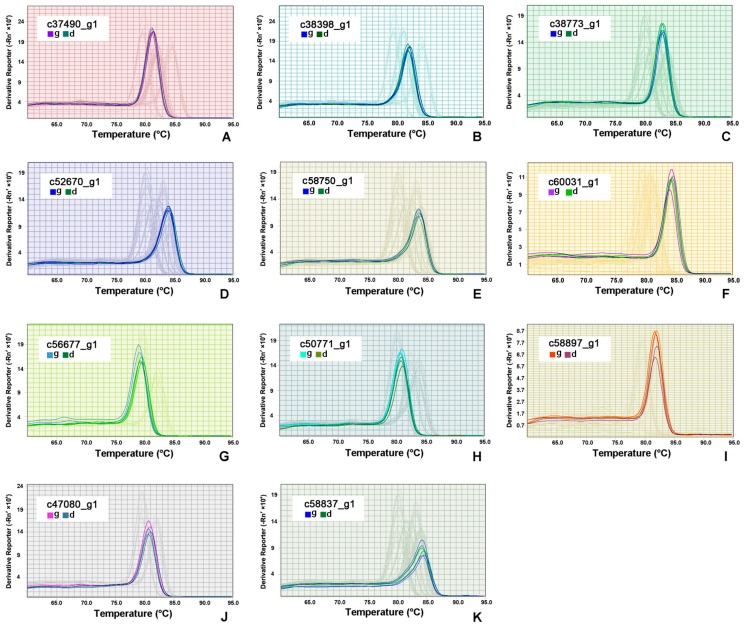
RT-qPCR melting curves for five up-regulated (**A**–**E**) and six down-regulated differentially expressed genes (**F**–**K**). (**A**), c37490_g1; (**B**), c38398_g1; (**C**), c38773_g1; (**D**), c52670_g1; (**E**), c60031_g1; (**F**), c60031_g1; (**G**), c56677_g1; (**H**), c50771_g1; (**I**), c58897_g1; (**J**), c47080_g1; (**K**), c58837_g1. In each figure, “g” and “d” represent stage G and stage D.

In order to validate results of transcriptome analyses, we randomly selected 6 down- and 5 up-regulated DEGs from the top 100 DEGs for RT-qPCR. Melting curve analysis of RT-qPCR demonstrated a single product for all tested DEGs (Figure 6). The highly up-regulated DEGs alpha-tubulin, 14-3-3 domain containing protein, histone variant H3.8, electron transferring flavor protein dehydrogenase, and proteasome subunit beta type were chosen from group D. Their expression levels were 1.74, 2.46, 6.64, 1.36, and 2.28 times higher, respectively, than those in group G (Table 3). When comparing group D with group G, the predicted DEGs eukaryotic aspartyl protease family protein (c60031_g1), EF hand family protein (56677_g1), cAMP-dependent protein kinase catalytic subunit (c50771_g1), citrate synthase (58897_g1), adenosine kinase (c47080_g1), and s-adenosyl-l-homocysteine hydrolase (c58837_g1) were down-regulated. The expression levels of these 6 DEGs in group D were decreased by 1.89, 6.61, 1.22, 3.68, 2.71, and 1.35 times, respectively, when compared to group G (Table 3). The log2-fold changes from RT-qPCR and RNA-Seq expression profiles were largely consistent (Figure 2B).

## 4. Discussion

### 4.1. Ciliates Share Essential Mechanisms of Cell Division with Other Eukaryotic Organisms

To identify the transcriptome profile of vegetative cell division and to control of the cell cycle progression of ciliates, we used single cells of *Pseudokeronopsis erythrina* at the G and D stages and performed RNA-Seq in triplicate for each stage. A combined annotation profile derived from COG, GO, and KEGG comprised unigenes related to cell division and growth, consistent with the cell stages being analyzed. Herein, 2051 predicted unigenes were differentially expressed between stages G and D. Hierarchical clustering (Figure 2A) of the expression profiles clearly separated the two stages, indicating that the transcribed unigenes in the two stages were rather different. Essential genes involved in the progression of cell division have been characterized in various organisms, including protists [6,7,8,52,53,54].

As expected, vegetative proliferation of *Pseudokeronopsis erythrina* shared many similarities with the cell division cycles of other eukaryotic organisms. In eukaryotes, the cell cycle comprises four separate phases: growth (G1), DNA synthesis (S), growth (G2), and mitosis (M). Cell cycle progression from G1-S-G2-M occurs via the action of CDKs. The latter are activated when they form complexes with their corresponding cyclins, which are synthesized and degraded throughout the cell cycle. Specific cyclins are expressed at various stages of the cycle. Both the regulation and order of progression are highly conserved across eukaryotes, including yeast, mammals, and plants [55,56,57,58]. Herein, transcribed sequences corresponding to CDKs and cyclins were mostly up-regulated during the D stage (Table 2), in agreement with previous studies [56,57,58]. Specifically, mitotic cyclins and their corresponding CDKs were highly up-regulated, while cyclins of the G1/S phase were down-regulated. This indicates that the cell was past the S and G2 phases and at the peak of mitosis, possibly at the end of metaphase and beginning of anaphase. In support of this, predicted unigenes corresponding to the anaphase-promoting complex and proteasome subunits were also highly up-regulated during the D stage (Table 2). Consistent with this finding, analysis of predicted DEGs revealed significant enrichment in the chromosome partitioning COG category and APC-associated subunits in both GO terms and KEGG pathways. The APC is an ubiquitin ligase that ensures progression of mitosis by mediating protein degradation of multiple targets including cyclins. This occurs by adding a ubiquitin tag to the protein, which is then targeted for degradation via the proteasome. APC becomes active once all chromosomes are attached to the mitotic spindle and thus ensures progression of cell division to anaphase [56]. Similar to other protists, yeasts and metazoans, future studies focusing on identifying CDK/cyclins complexes and associated proteins of various ciliates would be of interest [52,59,60].

Transcribed sequences corresponding to proteins with DYRK domains were strongly up-regulated, including dual specificity protein phosphatases. A putative ortholog of cdc14 phosphatase was also identified. This phosphatase has a regulatory role during anaphase in budding yeast; its observed significant upregulation noted herein suggests that it likely has a similar role in *P. erythrina*. Members of DYRKs phosphorylate a wide range of targets including cell cycle regulators. Previous studies have revealed that DYRK regulates cell proliferation [61] and phosphorylation of microtubule-organizing proteins [62].

Transcripts corresponding to MORN domain-containing proteins, MAPK, and tubulins, all of which are known to be closely related to cell proliferation, were significantly up-regulated during the D stage (Table 2). MORN domain-containing proteins have been proposed to regulate cellular localization and enzyme activity and stimulate vegetative growth [63,64,65,66]. MAPK genes also play important roles in the proliferation and differentiation of mammalian cells [67]. Tubulin genes are the major components of microtubule cytoskeletons in eukaryotic cells and are highly modulated in cell division [8]. The mitotic spindle, which forms during mitosis, is comprised of tubulin heterodimers. Up-regulation of transcripts associated with tubulin suggests that the cell elongation of *Pseudokeronopsis erythrina* at the D stage might occur through active tubulin extension, as demonstrated in the kinetoplastid *Trypanosoma* [59].

Overall, the ciliate *Pseudokeronopsis erythrina* shares many essential cell cycle pathways with other eukaryotic organisms, highlighting the conserved nature of this process. Previous transcriptome analyses detected homologs of yeast cell cycle regulators in dinoflagellate protists [52] while several components essential in yeast were absent. Given that single-cell transcriptomics cannot reliably capture low-abundance transcripts [68,69], it is too early at this stage to draw conclusions regarding absent components in *P. erythrina*. Future work should focus on deep sequencing using multiple cells so that the overall diversity of transcribed cell cycle components is captured.

### 4.2. Comparisons with Previous Studies Focusing on the Cell Cycle Regulation of Ciliates

Previous morphogenetic studies of ciliates [14,15,70,71,72] have shown that many biological processes, including somatic cortex morphogenesis, stomatogenesis, and organelle morphogenesis, occur at stage D. Transcriptomes from the G and D stages (Figure 4C) and RT-qPCR analyses revealed that phosphotransferase activity might be related to cortex modifications [73] as it was enriched during the D stage. Common amino acid residues typically phosphorylated by kinases include serine, threonine, and tyrosine. Our analyses showed that serine/threonine kinase activity was significantly enriched at the D stage (Supplementary Table S6), indicating that this kinase might also play a role in *Pseudokeronopsis erythrina* somatic cortex morphogenesis. Serine/threonine protein kinase and cation transport ATPase were also significantly enriched. Both of these fell under the GO term of development, indicating potential association with the formation of new oral structures (Supplementary Table S6). Several organelle-related biological processes were enriched (Figure 4A), in agreement with previous morphogenetic studies of *P. erythrina*, whereby the two “sister cells” (proter and opisthe) are created after organelle differentiation and dedifferentiation [15]. Previous transcriptome investigation of *Cryptocaryon irritans*, a parasitic ciliate species, also indicated that organelle-related biological processes were enriched during the population growth of ciliates [44]. Experiments using technologies such as gene silencing and fluorescence in situ hybridization are suggested to validate bioinformatic results.

Previous morphological and cell biological studies have indicated that vegetative division and cellular regeneration of ciliates share many similarities [74,75]. In line with this, most significantly enriched DEGs are involved in developmental, organelle, and microtubule-based processes. Previously, Onsbring et al. [76] studied the regeneration of *Stentor polymorphus* and identified several eukaryotic cell cycle-related biological processes and homologs known for their roles in eukaryotic cell division. Serine/threonine kinase at the D stage was most significantly enriched in our study (Figure 4C), in agreement with the results of Onsbring et al. [76], indicating the overlap of expressed genes in regeneration and cell division processes. Furthermore, MAPK genes, closely related to cell proliferation, were up-regulated at the D stage in *Pseudokeronopsis erythrina* (Supplementary Table S3), as well as at the alternative splicing stage in the conjugation cell cycle of *Tetrahymena thermophila* [9]. Herein, we showed that some molecular mechanisms are shared among vegetative division, conjugation, and cellular regeneration in ciliates, based on transcriptomes of vegetative division for the first time. Future studies should focus on the cell biology of *P. erythrina* to further characterize the transcribed sequences identified in this study.

Since vegetative cell division of ciliates has rich morphological patterns [14,28,40,43,77], we analyzed, for the first time, the mechanism of proliferation during the vegetative cell cycle in these organisms. Future studies geared towards ciliate species with different types of morphogenetic patterns will greatly enrich our knowledge of the molecular mechanisms of ciliate vegetative cell cycles and will facilitate better understanding of the proliferation process of eukaryotic cells overall.

## Figures and Tables

**Table 1:** Statistical summary of sequencing and assembly results.

Sample	D1	D2	D3	G1	G2	G3
raw read number	87,103,778	84,670,776	57,275,044	52,963,602	60,176,334	50,426,634
clean read number	39,375,244	40,285,356	41,369,504	41,684,596	43,051,906	35,788,890
raw base number (Mb)	12,460.30	12,112.25	8193.26	7576.50	8608.29	7213.59
clean base number (Mb)	5576.19	5721.74	5913.74	5958.98	6154.61	5116.24
Q30 (%)	81.4	82.25	96.37	96.39	96.13	96.27

**Table 2:** Significant up- or down-regulated expressed in stage D compared to stage G.

Gene Family	Up/Down	Gene_id	Nr Description
CYCs	up	c62398_g1	Mitotic cyclin-CYC2 [*Oxytricha trifallax*]
	up	c67538_g2	Cyclin [*Oxytricha trifallax*]
	up	c17611_g1	Cyclin-T [*Oxytricha trifallax*]
	up	c9656_g1	Cyclin, N-terminal domain-containing protein [*Oxytricha trifallax*]
	up	c36176_g1	Mitotic cyclin-CYC2, putative [*Oxytricha trifallax*]
	up	c48130_g1	Cyclin, N-terminal domain-containing protein [*Oxytricha trifallax*]
	up	c54046_g1	Amine-terminal domain cyclin [*Tetrahymena thermophila* SB210]
	down	c60024_g1	G1/S-specific cyclin-E1, putative [*Oxytricha trifallax*]
APCs	up	c57016_g1	TPR repeat-containing protein [*Oxytricha trifallax*]
	up	c75917_g1	TPR repeat-containing protein [*Oxytricha trifallax*]
	up	c54345_g1	Anaphase-promoting complex subunit 8-like [*Stylonychia lemnae*]
	up	c58189_g1	Anaphase-promoting complex subunit 10 [*Stylonychia lemnae*]
	up	c16402_g1	Anaphase-promoting complex subunit 11 [*Stylonychia lemnae*]
CDKs	up	c61357_g2	Cyclin-dependent kinase b2-2-like [*Stylonychia lemnae*]
	up	c40281_g1	Cyclin-dependent kinase regulatory subunit family protein [*Stylonychia lemnae*]
DYRK	up	c61235_g1	Putative dual specificity protein phosphatase cdc14 [*Oxytricha trifallax*]
	up	c58364_g1	Dual specificity protein phosphatase [*Stylonychia lemnae*]
	down	c47824_g1	Dual specificity phosphatase, catalytic domain-containing protein [*Oxytricha trifallax*]
	down	c12202_g1	Dual specificity catalytic domain-containing protein [*Stylonychia lemnae*]
MORN	up	c56401_g1	Putative MORN repeat protein [*Oxytricha trifallax*]
	up	c60320_g1	Putative MORN repeat protein [*Oxytricha trifallax*]
	up	c60776_g1	Putative MORN repeat protein [*Oxytricha trifallax*]
	up	c48198_g1	Putative MORN repeat protein [*Oxytricha trifallax*]
	up	c66123_g1	Putative MORN repeat protein [*Oxytricha trifallax*]
	up	c62710_g1	MORN repeat protein [*Stylonychia lemnae*]
	up	c60934_g1	MORN repeat protein [*Stylonychia lemnae*]
	up	c50818_g1	MORN repeat-containing protein 5 [*Stylonychia lemnae*]
	up	c59470_g1	MORN repeat protein [*Stylonychia lemnae*]
	up	c26899_g1	MORN motif protein [*Tetrahymena thermophila* SB210]
MAPK	up	c57299_g1	Putative MAPK [*Oxytricha trifallax*]
	up	c59004_g1	Mitogen-activated protein kinase kinase [*Oxytricha trifallax*]
Tubulin	up	c37490_g1	Alpha tubulin, putative [*Oxytricha trifallax*]
	up	c7781_g1	Microtubule-associated RP/EB member 2 [*Stylonychia lemnae*]

**Table 3:** Up- or down-regulated DEGs checked by RT-PCR.

Gene id	Nr Description	Up/ Down	Fold Change	log2 Fold Change
c37490_g1	Alpha tubulin, putative [*Oxytricha trifallax*]	up	3.34	1.74
c38398_g1	14-3-3 domain-containing protein [*Oxytricha trifallax*]	up	5.5	2.46
c38773_g1	Histone variant H3.8 [*Stylonychia lemnae*]	up	100.01	6.64
c52670_g1	Electron transferring flavor protein dehydrogenase [*Oxytricha trifallax*]	up	2.56	1.36
c58750_g1	Proteasome subunit beta type [*Stylonychia lemnae*]	up	4.87	2.28
c60031_g1	Eukaryotic aspartyl protease family protein [*Stylonychia lemnae*]	down	0.27	−1.89
c56677_g1	EF hand family protein [*Stylonychia lemnae*]	down	0.01	−6.16
c50771_g1	cAMP-dependent protein kinase catalytic subunit, putative [*Oxytricha trifallax*]	down	0.43	−1.22
c58897_g1	Citrate synthase [*Stylonychia lemnae*]	down	0.09	−3.68
c47080_g1	Adenosine kinase [*Stylonychia lemnae*]	down	0.22	−2.17
c58837_g1	S-adenosyl-L-homocysteine hydrolase [*Stylonychia lemnae*]	down	0.39	−1.35

## Supplementary Materials

Supplementary materials can be found at https://www.mdpi.com/2076-2607/8/1/108/s1. Table S1 primer sequences in the RT-qPCR experiment; Table S2 summary of de novo assembly of transcriptomic profiles of *Pseudokeronopsis erythrina*; Table S3 detailed information of DEGs between stages G and D; Table S4 COG annotation of DEGs between stages G and D; Table S5 COG analysis of DEGs in cell cycle control, cell division, and chromosome partitioning category; Table S6 GO enrichment analysis of DEGs in the biological process, cell components, and molecular function category; Table S7 KEGG pathway enrichment analysis of DEGs.
