# Supplementary material for: Comparative Transcriptome Analyses during the Vegetative Cell Cycle in the Mono-Cellular Organism Pseudokeronopsis erythrina (Alveolata, Ciliophora)
Source: Microorganisms. 2020 Jan 12;8(1):108. doi: 10.3390/microorganisms8010108 (PMC7022673; doi:10.3390/microorganisms8010108)
Supplement: Supplementary file 1 [file microorganisms-08-00108-s001.zip › microorganisms-642101-supplymentary/Table S1.pdf]

Table S7 DEGs primers

Gene_id	Sequence (5'-3')	Tm	PCR product (bp)
c1293_g1	ATCCTGTTTGCACGCTTTGG<br>GGATGCATATGTGGCCCTGA	60	128
c37490g1	CTGTTCGTGGTACGCCTTCT<br>GATGGTGCCCTGAATGTGGA	60	123
c38398_g1	GTTGGCAGGCTTTCTTGTGG<br>GAAGCAGGAGGCTCTGAAGG	60	140
c38773_g1	CCACGCAAACAGATCAACCA<br>ATCTTGAGCCGTGAAGGTCC	60	126
c52670_g1	ACCCAACTTGTCCCTCCCTT<br>GCAGCCTTACCAACTCCATG	60	155
c58750_g1	CTGCGGAGTAATCTGGAAGG<br>AGATGTTCGGAGCGAGGTAG	60	116
c60031_g1	GGAATCCGAGGTAGCAGACG<br>GCTGACTCCGACCAAGTGAA	60	190
c56677_g1	GGGATTTTGCGCCTTATTC<br>TTCCGACACTCCCACCATT	60	116
c50771_g1	AGCGCCCAATAATCCACTCC<br>TAGGGACTGACGGCCATTTG	60	151
c58897_g1	AGACGGAGCCATCAATGCAA<br>AAATGCAGGCAACCGTGTTC	60	104
c47080_g1	TTGGCAAGGACGAGTATGG<br>CAAGGAAGTGCAAAGTGGG	60	183
c58837_g1	TGATGTCGGAAAGGGATGC<br>CGGTAGCGGTCACGAAAAT	60	154
