# Supplementary material for: Comparative Transcriptome Analyses during the Vegetative Cell Cycle in the Mono-Cellular Organism Pseudokeronopsis erythrina (Alveolata, Ciliophora)
Source: Microorganisms. 2020 Jan 12;8(1):108. doi: 10.3390/microorganisms8010108 (PMC7022673; doi:10.3390/microorganisms8010108)
Supplement: Supplementary file 1 [file microorganisms-08-00108-s001.zip › microorganisms-642101-supplymentary/Table S2.pdf]

Table S1 Summary of the de novo assembly of transcriptomic profiles of *P. erythrina*

	Total length (bp)	Count	Max length (bp)	Average length (bp)	N50	GC %
Trinity	58,260,420	88,014	21,889	661.94	880	43.83
Unigene	47,567,028	76,358	21,889	622.95	806	44.59
